# The Cross-Cultural Adaptation, Validation and Psychometric Properties of the Mental Fatigue Scale in Turkish Athletes

**DOI:** 10.3390/brainsci16010074

**Published:** 2026-01-06

**Authors:** Yusuf Soylu, Leonardo de Sousa Fortes, Ersan Arslan, Haitham Jahrami, Bulent Kilit, Khaled Trabelsi, Achraf Ammar, Jesús Díaz-García

**Affiliations:** 1 Faculty of Sports Sciences, Tokat Gaziosmanpasa University, Tokat 60250, Türkiye; ersan.arslan@gop.edu.tr (E.A.); bulent.kilit@gop.edu.tr (B.K.); 2 Department of Physical Education, Universiade Federal da Paraíba (UFPB), Joao Pessoa 58051-900, Brazil; leodesousafortes@hotmail.com; 3 Department of Psychiatry, Ministry of Health, Manama 410, Bahrain; haitham.jahrami@outlook.com; 4 Department of Psychiatry, College of Medicine and Medical Sciences, Arabian Gulf University, Manama 323, Bahrain; 5 Department of Movement Sciences and Sports Training, School of Sport Science, The University of Jordan, Amman 11942, Jordan; trabelsikhaled@gmail.com; 6 Research Laboratory, Molecular Bases of Human Pathology, LR19ES13, Faculty of Medicine of Sfax, University of Sfax, Sfax 3000, Tunisia; acammar@uni-mainz.de; 7 Research Laboratory, Education, Motricity, Sport and Health, EM2S, LR19JS01, High Institute of Sport and Physical Education of Sfax, University of Sfax, Sfax 3000, Tunisia; 8 Department of Training and Movement Science, Institute of Sport Science, Johannes Gutenberg-University Mainz, 55122 Mainz, Germany; 9 Faculty of Sport Sciences, University of Extremadura, 06006 Caceres, Spain; jdiaz@unex.es

**Keywords:** cross-cultural studies, fatigue, cognitive fatigue, reliability, scale adaptation, mental health

## Abstract

**Background/Objective**: This study aimed to adapt the Mental Fatigue Scale (MFs) to evaluate the psychometric properties in adult and adolescent athletes. **Methods**: A total of 491 adolescent and adult athletes (n = 491) consisting of 204 adults (men = 115; female = 90; age = 24.38 ± 3.18 year) and 287 adolescents (men = 178; female = 109; age = 14.97 ± 1.55 year) who actively participated in various sports branches voluntarily participated in this study. The MFs consists of fifteen (15) items and a single-factor structure and is a measurement tool used to measure the general mental fatigue level of athletes. Two experts used a four-point Likert scale to assess the content validity of each of the fifteen MFs items, which were aligned with the provided definition of mental fatigue in a sports context. Drawing on these findings, a confirmatory factor analysis was conducted on the survey data collected to assess the construct validity of this measure. **Results**: The outcomes of the confirmatory factor analysis provided acceptable support for factorial validity (χ^2^/sd = 1.52; *p* < 0.01, SRMR = 0.05, RMSEA = 0.08, GFI = 0.94, CFI = 0.89, NNFI = 0.87). Additionally, multi-group confirmatory factor analysis supported measurement invariance, indicating that the scale functions equivalently across adolescent and adult athletes. Furthermore, the analysis demonstrated favorable internal consistency (α = 0.88), confirming the reliability of the MFs. Test–retest after two weeks revealed an intra-class correlation of 0.90. **Conclusions**: Collectively, these results suggest that the MFs is a dependable and valid instrument that is particularly valuable for gauging overall mental fatigue in athletes. Coaches and sports scientists can use this assessment tool to evaluate athletes’ general mental fatigue effectively.

## 1. Introduction

Mental fatigue is conceptualized in the present study as a distinct psychobiological state characterized by reduced cognitive efficiency, impaired executive functions, and an increased perception of effort, which emerges following prolonged or intense cognitive demands [1,2]. Unlike physical fatigue from muscle activity and metabolic depletion, mental fatigue stems from central mechanisms arising from cognitively demanding tasks [3]. Moreover, mental fatigue must be distinguished from mental effort, which refers to the amount of cognitive resources invested during task execution, rather than the cumulative psychobiological state that develops over time [4]. However, athletes often face challenges in differentiating between the sensations of fatigue and effort [5]. Recent evidence suggests that perceived effort may act as an indirect indicator or confounding factor in fatigue assessment, highlighting the necessity for multidimensional scales, such as the Mental Fatigue Scale (MFs), that can capture specific symptoms beyond general tiredness [6].

Mental fatigue is often caused by sustain or intense high cognitive load tasks and is accompanied by feelings of tiredness [1,2]. In the sports context, additional sources such as intensive training, competitions, and travel demand significantly contribute to the indication of mental fatigue [7]. Indeed, in athletic contexts, overall performance depends not only on physical conditioning but also on cognitive capacity and the ability to resist mental fatigue [8]. Therefore, distinguishing and specifically measuring the mental component of fatigue, separate from physical tiredness, is essential for a holistic understanding of athletic performance. Previous studies have demonstrated that this distinction is crucial because mental fatigue substantially impairs the decision-making processes essential for optimal performance [9,10]. Experimental studies have demonstrated negative effects on attentional control, visual search behavior, and technical execution in sports such as soccer and basketball [11,12,13]. Moreover, mental fatigue has been reported to decrease an individual’s ability to inhibit responses and process information [14], which is vital for athletes to resist impulsive actions during play. Furthermore, Schampheleer and Roelands [15] pointed out that the cumulative effects of fatigue not only reduce physical power output but also hinder critical decision-making capabilities. Although physiological markers, such as reduced prefrontal oxyhemoglobin concentration or increased theta-band activity, can objectively detect these states [16,17], these measures require specialized equipment (fNIRS, EEG) and personnel. Consequently, subjective scales remain the most practical and cost-effective solution for routine monitoring in sports settings.

The Visual Analogue Scale (VAS) is one of the most frequently used tools in sports studies for measuring acute mental fatigue [11,12,18]. Although the VAS is widely used for its simplicity, it has methodological disadvantages, such as unstandardized anchoring and poor metacognitive control, leading to discrepancies between subjective perception and objective states [14,19]. Kunasegaran et al. [14] highlighted that subjective measurement tools are often criticized for being susceptible to response biases, as the introspective abilities of individuals can be easily compromised. Furthermore, evidence supporting the validity of the VAS, specifically in athletic samples, remains limited [14,20]. Therefore, unlike acute state measures such as the VAS, which assess momentary fatigue, athletes frequently encounter the cumulative effects of both physical and mental demands over the course of their training cycles [21]. Pageaux and Lepers [22] indicated that such accumulation can lead to chronic disorders in performance and well-being. Single-dimensional tools generally require a multi-dimensional tool, such as MFs, to accurately assess an athlete’s condition because of their disregard for complexity. Moreover, Lismane et al. [6] highlighted the limitations of using psychometrically unvalidated unidimensional tools, which may lead to an inaccurate assessment of an athlete’s actual cognitive state [6]. Thus, in contrast to single-item scales, a multidimensional tool such as the MFs, which captures a broader symptomatology beyond a single time point, is necessary [14].

Despite current evidence, there is a lack of psychometrically sound and culturally adapted tools to assess mental fatigue in the sports environment. The MFs was originally developed by Johansson et al. [23] to assess mental fatigue and related symptoms following neurological disorders. It is a multidimensional self-report instrument consisting of 15 items that evaluate affective, cognitive, and sensory symptoms, as well as sleep duration and daytime variability. Although the scale has demonstrated validity in clinical populations and has recently been adapted for healthcare professionals [24], its psychometric properties have not yet been established in an athletic context. Although mental fatigue is increasingly being investigated in sports, most studies rely on tools that are not culturally adapted or validated for athletes, restricting the generalizability of the findings [25]. Moreover, Kunasegaran [14] stated that monitoring and early recognition play critical roles in preventing acute symptoms from transitioning into chronic fatigue conditions. In the Turkish sports context, the absence of a psychometrically validated instrument limits practitioners’ ability to accurately monitor mental fatigue and optimize training loads. Therefore, the primary aim of the present study was not to develop a new instrument but to adapt and validate the existing MFs for use in Turkish-speaking adolescent and adult athletes and to examine its psychometric properties within this population.

## 2. Materials and Methods

### 2.1. Participants

The study group (n = 491) consisted of 204 adults (men = 115; female = 90; age = 24.38 ± 3.18; height = 175.31 ± 8.23; weight = 67.21 ± 10.69; body mass index = 21.81 ± 2.70; athletic experience = 9.86 ± 4.39; national athlete = 26) and 287 adolescents (men = 178; female = 109; age = 14.97 ± 1.55; height = 168.53 ± 10.90; weight = 58.61 ± 12.00; body mass index = 20.55 ± 4.20; athletic experience = 5.05 ± 2.80; national athlete = 12) who were actively or recreationally involved in different sports (football, boxing, fencing, basketball, volleyball, kickboxing, handball, taekwondo, table tennis, karate, wushu, wrestling, track and field, field hockey, badminton, tennis, orienteering, and archery). Participants were recruited from sports clubs and university sports programs in Türkiye. Recruitment was conducted through coaches, physical education instructors, and sports administrators who informed the athletes about the study objectives. A convenience sampling method, which is common in psychometric validation studies, was used. Sampling methods and participant characteristics were reported to evaluate generalizability, following guidelines [26]. Participation was voluntary, and no incentives were offered. The inclusion criteria were as follows: (a) active, recreational, amateur, or professional athletes engaged in organized sports training; (b) aged between 13 and 30 years; and (c) sufficient Turkish proficiency to complete the questionnaire. The exclusion criteria were as follows: (a) self-reported neurological disorders, psychiatric diagnoses, or cognitive impairments that could influence mental fatigue perception and (b) incomplete questionnaire responses. All participants and their respective parents or guardians provided written informed consent prior to the commencement of this study, in accordance with the principles outlined in the Helsinki Declaration. This study was approved by the Research Ethics Committee of Tokat Gaziosmanpasa University (Approval date 30.05.2023, Approval No. 01-16).

### 2.2. Procedures

Translation and Cultural Adaptation: To adapt the scale to the Turkish population, permission was obtained from the original author, Birgitta Johansson. The adaptation process followed the established guidelines for the cross-cultural adaptation of self-report instruments [27,28]. The process involved the following steps, including forward translation, which was conducted independently by two bilingual translators whose native language was Turkish. One translator was informed of the study concepts, while the other was blinded to the study hypothesis to ensure diverse linguistic expressions. The synthesized Turkish version was back-translated into English by two bilingual translators who were native English speakers and fluent in Turkish. Crucially, these translators were blinded to the original scale and study objectives to avoid bias and ensure conceptual accuracy. An expert committee comprising researchers in sports science, exercise psychology, and psychometrics reviewed all versions (original, forward, and backward translations). This step was critical to ensure not only linguistic equivalence but also semantic, idiomatic, and conceptual equivalence between the original and Turkish versions. Minor linguistic modifications were made to improve clarity in the sports context. Although the expert committee ensured content validity, a formal cognitive debriefing or pilot testing with the target population was not conducted prior to full administration. This is acknowledged as a methodological limitation of this study. However, the factorial structure and internal consistency of the adapted version were subsequently examined using confirmatory factor analysis (CFA) in the target athletic population (n = 491) to provide statistical evidence of its construct validity.

Data Collection: Following the adaptation process, data collection was conducted between 6 April 2024 and 4 May 2024. Participants were recruited from sports clubs and universities. The questionnaires were administered via an online platform and using printed forms in a quiet environment to minimize the distractions. The approximate time to complete the MFs was 5–10 min. To ensure that the assessment reflected general mental fatigue rather than acute exhaustion, data were collected on rest days. Participants were briefed about this study’s anonymity and voluntary nature prior to participation.

### 2.3. Measurements

The MFs is a multidimensional self-assessment instrument developed by Johansson et al. [23] and adapted from Rödholm et al. [29]. The 15 items cover emotional, cognitive, and sensory symptoms, including sleep duration and daytime variations. Each item includes common examples of activities to ensure consistent assessment. Responses are rated on a scale of 0–3 (0 indicates normal function, 1 indicates a problem, 2 indicates noticeable symptoms, and 3 indicates maximum symptoms). Half-point ratings (0.5, 1.5, and 2.5) were permitted for finer discrimination. Symptom intensity, frequency, and duration were integrated into a single rating selection and not scored separately. Higher scores indicate more intense, frequent, and enduring symptomatology. The total score is calculated by summing the items (typically the first 14 items, with item 15 providing descriptive information on the diurnal variation). In the original validation study, a cut-off score of 10.5 was established to significantly distinguish between patients with neurological disorders and healthy controls, serving as the threshold for identifying significant mental fatigue [23].

### 2.4. Statistical Analyses

Descriptive statistics were used to describe the sample characteristics and summarize the results. The mean was used as a measure of central tendency, and the standard deviation was used as a measure of the variability. Frequencies and percentages were used to describe the distribution of responses for categorical variables. We compared the MFs scores between adults and adolescents. We used independent sample t-tests to assess the differences in the means between these groups.

The test–retest reliability of Turkish MFs was assessed using the intraclass correlation coefficient (ICC). To evaluate temporal stability, a randomly selected subset of participants (n = 120; consisting of 60 adults [30 male, 30 female] and 60 adolescents [30 male, 30 female]) re-completed the scale. This specific timeframe was chosen to minimize recall bias regarding previous answers while ensuring construct stability over time. An ICC (two-way random-effects model, absolute agreement) was calculated. Following established guidelines, ICC values less than 0.50 were considered poor, 0.50–0.75 moderate, 0.75–0.90 good, and greater than 0.90 excellent [30].

The internal consistency of the Turkish version of the mental fatigue was assessed using McDonald’s omega and Cronbach’s alpha [31]. An internal consistency value > 0.70, which is widely regarded as an acceptable level of consistency [32,33], was set as the threshold for the analysis. We also examined whether any items that had weak correlations with other items during the analysis could be excluded from the questionnaire. However, no items were eliminated from this study because all items showed high internal consistency, suggesting that they all measured the same construct and enhanced the validity of the questionnaire. Test–retest reliability was examined using the ICC. The Turkish version of the MFs factor structure was investigated using confirmatory factor analysis (CFA) [34]. Prior to conducting the CFA, an exploratory factor analysis (EFA) was performed on the full sample (N = 491) to examine the underlying structure of the Turkish MFs and inform the subsequent confirmatory approach. Using maximum likelihood extraction with promax rotation, the number of factors to retain was determined based on a parallel analysis, inspection of the scree plot, and theoretical expectations of unidimensionality. The EFA supported a single-factor solution, with all 15 items loading substantially on one factor (loadings ranging from 0.42 to 0.69) and explaining 34% of the variance. This preliminary evidence of unidimensionality justified the testing of a one-factor model in the CFA. CFA is a statistical technique that enables researchers to examine the degree to which observed data conform to a proposed theoretical framework [34]. In this study, a one-factor model, which postulates that all questionnaire items measure the same construct, was hypothesized [34].

The data were examined using structural equation modelling (SEM) to conduct a CFA [21]. The maximum likelihood estimation method, an often-employed technique for SEM, was used to conduct the CFA. The comparative fit index (CFI), Tucker–Lewis index (TLI), root mean square error of approximation (RMSEA), and Standardized Root Mean Square Residual (SRMR) were the fit indices used to assess the model’s goodness of fit SRMR [35]. These indices were chosen to provide a comprehensive evaluation of the model fit, encompassing both absolute fit (e.g., RMSEA and SRMR, which assess how well the model reproduces the observed covariance matrix) and incremental fit (e.g., CFI and TLI, which compare the proposed model to a baseline model) [33]. Acceptable fit was indicated by CFI and TLI values ≥ 0.90, RMSEA ≤ 0.08 (with 90% CI upper bound < 0.10), and SRMR ≤ 0.08 [34,36]. Measurement invariance across adolescent and adult athletes was examined using multi-group confirmatory factor analysis (MG-CFA) with progressively constrained models: configural (same factor structure), metric (equal factor loadings), scalar (equal item intercepts), strict (equal residual variances), and structural (equal factor variances) models. Model comparisons revealed minimal changes in the fit indices (ΔCFI = 0.00, |ΔRMSEA| ≤ 0.01, ΔSRMR = 0.00), supporting configural, metric, scalar, and strict invariance. The suite for statistical computation and visualization offered by the R Statistical Foundation (version 4.2.2) was used for all analyses. The threshold for statistical significance in all analyses was set at *p* ≤ 0.05.

## 3. Results

The sample size was 491, and the mean age was 18.88 years, with a standard deviation of 5.5. The mean height and weight were 171.35 cm and 61.98 kg, respectively. The mean athletic experience was 7.05 years. The mean scores for the MFs items ranged from 0.36 to 1.15. The mean BMI was 21.07, with high skewness and kurtosis values. Table 1 provides a comprehensive overview of the participants’ characteristics of this study. Table 1 provides a detailed description of the skewness and kurtosis of the 15 MFs items.

McDonald’s omega test was used to determine scale accuracy. The result was 0.88 (95%CI 0.87–0.90), indicating an acceptable level of internal consistency. Cronbach’s alpha was also calculated, and the resultant value was identical to that of McDonald’s omega 0.88 (95%CI 0.87–0.90), further confirming the scale’s acceptable level of internal consistency. No items were suggested for deletion to improve McDonald’s omega or Cronbach’s alpha. The detailed results are listed in Table 2. Temporal stability was assessed in a randomly selected subsample of 120 participants who completed the scale twice at a two-week interval. The analysis yielded an ICC of 0.90 (95% CI: 0.88–0.92), indicating excellent test–retest reliability.

Exploratory factor analysis revealed a clear one-factor structure for Turkish MFs. The single factor accounted for 34% of the variance (eigenvalue = 5.78 pre-rotation; sum of squared loadings = 5.14 post-rotation), with all 15 items demonstrating salient loadings on this factor (range: 0.42–0.69 after the promax rotation). The highest-loading items were MF_5 (0.69), MF_1 (0.67), and MF_2 (0.66), while the lowest were MF_13 (0.42) and MF_14 (0.43). All items exceeded the conventional threshold of 0.30, supporting their retention. Uniqueness values ranged from 0.53 to 0.82, indicating adequate common variance captured by this factor. These EFA results aligned closely with the subsequent confirmatory factor analysis, which also confirmed a unidimensional model with acceptable fit indices (χ^2^/sd = 1.52, SRMR = 0.05, RMSEA = 0.08, GFI = 0.94, CFI = 0.89, NNFI = 0.87) and significant standardized loadings (range: 0.33–0.55).

As shown in Table 3, the confirmatory factor analysis yielded a CFI of 0.89 and a TLI/NNFI of 0.87. These values fell slightly below the traditional cut-off criteria (≥0.90) [36]. The NFI value was 0.85, which was below the strict threshold. However, recent methodological research cautions against the rigid application of universal cut-offs, particularly for single-factor models, where incremental fit indices can be attenuated [36,37]. Importantly, the absolute fit indices indicated a better fit, with an RMSEA of 0.08 and an SRMR of 0.05, which were within acceptable limits. Collectively, these indices indicate a borderline to acceptable model fit, supporting the factorial validity of the scale in this specific athletic population [33].

The results of the factor invariance analysis showed no significant differences between adults and adolescents in the factor structure of the scale, indicating that the Turkish MFs were valid for both populations. This finding suggests that MFs can be used to measure mental fatigue in adults and adolescents. Factor invariance analysis is an important step in establishing scale validity across populations. The results of this study provide evidence of the cross-population validity of MFs.

Table 4 provides important information regarding the psychometric properties of Turkish MFs and the strength of the relationship between each item and the underlying construct of mental fatigue. The table reports the item loading, standard error, z-value, *p*-value, and 95% confidence interval (CI) for each of the 15 items of the MFs. Item loading represents the strength of the relationship between each item and the underlying construct of mental fatigue. The results showed that all 15 items had positive and statistically significant loadings ranging from 0.33 to 0.55 (standardized loadings: 0.42 to 0.69). The standard errors were relatively small, indicating precise parameter estimates for the model. The z-values were large and statistically significant, indicating that the parameter estimates were significantly different from the zero. The 95% CIs were relatively narrow, indicating that the parameter estimates were precise and reliable.

**Figure 1:**
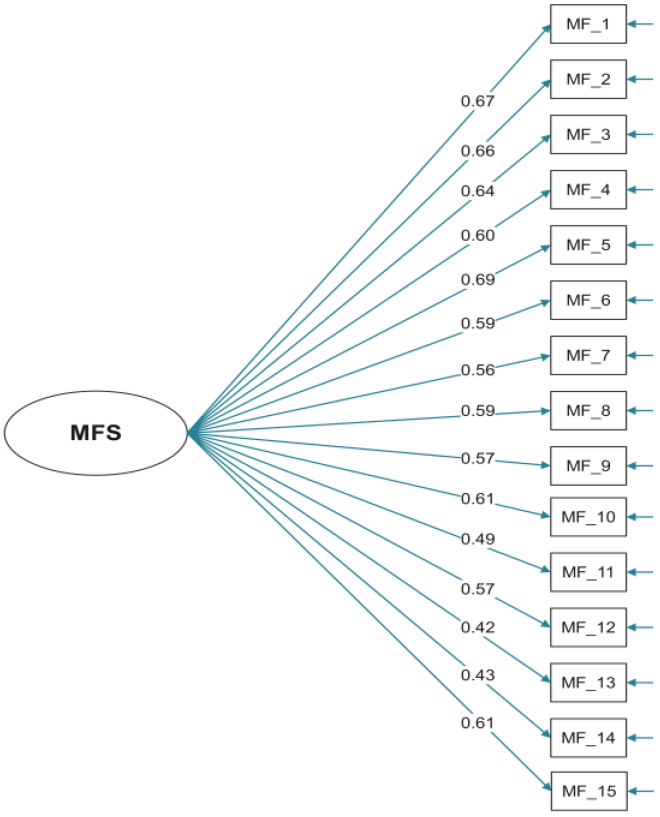
Path diagram for MFs.

In Figure 1, the standardized factor loadings for all 15 items were statistically significant (range: 0.42–0.69). Based on these loadings, Composite Reliability (CR) and Average Variance Extracted (AVE) were calculated to assess convergent validity. The CR value was 0.79, which is well above the recommended threshold of 0.70, indicating good construct reliability. The AVE value was 0.21.

Measurement invariance testing among adolescent and adult athletes demonstrated support for configural, metric, and scalar invariance, as evidenced by minimal changes in the fit indices across progressively constrained models (ΔCFI ≤ 0.01; ΔRMSEA ≤ 0.01; ΔSRMR ≤ 0.01). Specifically, the Comparative Fit Index (CFI) remained stable at 0.88 from configural to scalar models, the Tucker–Lewis Index (TLI) improved slightly from 0.86 to 0.88, and the RMSEA decreased from 0.08 to 0.07, with the SRMR consistent at 0.06. Although strict invariance (constraining residual variances) exhibited an acceptable fit (CFI = 0.88, RMSEA = 0.07), the structural model (constraining factor variances) also maintained a good fit. These findings confirm that the Turkish MFs possess an equivalent factor structure, item loadings, and intercepts across age groups, thereby facilitating valid mean comparisons of mental fatigue between adolescent and adult athletes (Table 3).

## 4. Discussion

The present study shows that the Turkish version of the MFs demonstrates a unidimensional structure and satisfactory reliability when applied to adolescent and adult athletes. These findings suggest that mental fatigue, as operationalized by MFs, can be meaningfully assessed in Turkish-speaking athletic populations. Traditionally, mental fatigue has been examined in clinical or occupational contexts, whereas its assessment in sports has relied on task-based measurements. This adaptation extends the theoretical applicability of the MFs by supporting its use as a trait-like self-report instrument in athletes, capturing the cognitive, emotional, and sensory aspects of mental fatigue beyond the acute task-induced state of mental fatigue. Johansson et al. [23] developed the original scale to assess mental fatigue and related symptoms following neurological diseases and injuries. MFs include established items that commonly include neurological injuries or diseases [29,38,39], such as sleep, sensory perception, emotions, and cognitive function. The updated scale incorporates novel inquiries concerning mental recuperation and fluctuations over 24 h. Within this measurement landscape, MFs provide a complementary approach by assessing the cognitive, emotional, and sensory symptoms that develop over time. Originally designed for clinical populations [23], MFs capture aspects of mental fatigue beyond acute task-induced states, including difficulties in concentration, mental lethargy, and sensitivity to stimuli [29,40,41,42]. Subjective assessments should be supplemented with objective measurement. However, a recent study [6] showed that subjectively reported fatigue parameters do not consistently predict objective cognitive decline after ultra-endurance events in healthy individuals. Although athletes reported mental and physical fatigue, these were not associated with executive functioning deficits. Nevertheless, sleep quality and perceived exertion were stronger predictors of cognitive changes. This complexity underscores the utility of employing MFs, which encompass sleep, sensory sensitivity, and cognitive symptoms, thereby offering a more comprehensive profile than unidimensional ratings that may overlook the distinction between perceived effort and mental fatigue. These findings suggest that this symptom-oriented framework can be meaningfully applied to athletes, extending the theoretical and practical relevance of MFs in sports.

Although no MFs has been specifically developed and validated for athletic populations, mental fatigue has been widely examined in sports science using various assessment methods. Previous studies have predominantly relied on state-based measures, such as single-item ratings or visual analog scales, to quantify acute mental fatigue following cognitive tasks or prolonged competitions [12,43,44]. Although these instruments have shown sensitivity to transient changes in mental fatigue, they mainly capture momentary perceptions rather than broader symptom profiles of accumulated mental fatigue. Moreover, Díaz-García et al. [20] noted that unidimensional scales (e.g., VAS) inadequately capture the complexity of mental fatigue and that existing valid tools are predominantly found in clinical settings, leaving a gap in the sports context. This study highlights that, despite the predominance of subjective scales (76%) in mental load assessment, the adaptation of a personalized and multidimensional instrument (e.g., MFs) is critical for evaluating the effects of fatigue and symptom profiles. Compared to single-item measures of mental fatigue, the MFs provide a multidimensional symptom profile, which is particularly relevant in sports settings with cognitive load, such as intensive training or a congested competition schedule. In addition to state-based mental fatigue measures, cognitive load instruments such as the NASA Task Load Index have been used in sports and exercise contexts to assess the mental demands of task complexity and attention [45]. However, cognitive load measures focus on perceived task difficulty rather than fatigue symptoms and reflect a construct that is distinct from mental fatigue [14,46]. Similarly, workload monitoring tools such as ratings of perceived exertion (RPE) and session-RPE are widely used in applied sports settings, but these scales predominantly reflect physical and physiological strain, with mental fatigue considered only indirectly [47]. To the best of our knowledge, no standardized scale has been specifically designed to measure mental fatigue in athletes. The MFs presented adequate CFA to verify the one-factor solution. The fit indices suggested that the one-factor model had an acceptable fit (χ^2^/sd = 1.52; *p* < 0.01, SRMR = 0.05, RMESA = 0.08, GFI = 94; CFI = 0.89, NNFI = 0.87). However, the findings provide psychometric support for the Turkish version of the MFs, showing a unidimensional structure, satisfactory factor loadings, and strong internal consistency (α = 0.88). The standardized item loadings (0.33–0.55) indicated a meaningful contribution to mental fatigue, consistent with the symptom-based conceptualization of the original scale development [23]. Regarding model fit, the CFI (0.89) and NNFI/TLI (0.87) values fell slightly below the conventional cut-off criteria (≥0.90). However, methodological literature suggests that strict cut-off values should not be applied mechanistically, particularly in single-factor models with large heterogeneous samples [36,37]. Importantly, other absolute fit indices such as RMSEA (0.08) and SRMR (0.05) were within acceptable limits, indicating that the model residuals were low and the data fit the model structure adequately. These findings suggest that although the model is not perfect, it possesses sufficient factorial validity for use in athletic populations. The AVE for the Turkish MFs was 0.21, below the 0.50 threshold, while the Composite Reliability (CR) was 0.79, exceeding 0.70. According to Fornell and Larcker [48], CR values above 0.60 indicate adequate convergent validity, even with a low AVE, which is common for complex psychological constructs. This shows that the scale reliably measures mental fatigue despite the lower variance at the item level. The results of this investigation are consistent with the nurse version of the MFs [24] for measuring mental fatigue in adult and adolescent athletes. Previous studies in healthcare populations have reported a reduced 12-item version of the MFs derived from the original 14 evaluative items [24]. In contrast, in the present study, confirmatory factor analysis was conducted on all 15 items of the original scale (including the 24 h variation item), and all items met the predefined criteria and were retained without any modification. In contrast to the sports context, considering internal consistency, the results showed that the scale had good reliability, with a Cronbach’s alpha of 0.88 in this study. According to established guidelines, coefficients above 0.70 are considered acceptable, values above 0.80 are preferred, and those exceeding 0.90 is regarded as excellent [49,50]. This value is comparable to that found in the original study that measured the psychometric properties of the original MFs [16]. Güven et al. [24] also found similar results in their Turkish adaptation study of the MFs scale on nurses. A brief mental fatigue questionnaire was developed and validated for healthy subjects and patients with muscular disease, CFS, recovered CFS, and depression. The questionnaire has good internal consistency and reliability, meaning that the items measure a similar construct, and that responses to the questionnaire are consistent over time. The brief MFs had a Cronbach’s alpha value of 0.85 for data from non-clinical subjects and 0.93 when clinical subjects were included [51]. The questionnaire effectively distinguished between patients with depression and CFS and normal participants, patients with muscle disease, and recovered patients with CFS. The MFs is grounded in a symptom-based view of mental fatigue as a multidimensional set of cognitive, emotional, and sensory symptoms rather than a transient task-induced state [23]. This perspective is consistent with cognitive-energetic frameworks of fatigue, which suggest that sustained mental demands lead to decreased attention, slowed processing, and increased perceived effort [2,52]. From a sports psychology perspective, this aligns with the psychobiological model of fatigue, which emphasizes the role of perception, motivation, and cognitive control in fatigue and its behavioral consequences [53]. Rather than directly measuring performance decrements, MFs capture subjective symptoms linked to altered effort regulation and attentional capacity. These findings extend the theoretical frameworks to athletic populations by demonstrating that mental fatigue symptoms form a coherent construct in adolescent and adult athletes. Support for measurement invariance across age groups suggests that the underlying structure of mental fatigue is interpreted similarly across different developmental stages. The high temporal stability in the test–retest analysis indicates that MFs capture stable aspects of mental fatigue, complementing state-based measures used in sports settings. Similarly, Hamann and Castengerdes [16] reported that mental fatigue is a state of arousal between wakefulness and sleepiness. However, the Fatigue Instantaneous Self-Assessment (F-ISA) scale is one-dimensional, short, practical, valid, and reliable for measuring mental fatigue and measures mental fatigue related to any task instead of general fatigue [16]. The Cronbach’s alpha value of the current MFs in the adaptation study on athletes was similar to that reported in previous studies. Therefore, the MFs can be considered a valid instrument for measuring mental fatigue in adult and adolescent athletes. Specifically, the factor invariance analysis revealed no significant differences in the factor structure between these groups. This indicates that mental fatigue is perceived similarly across developmental stages, allowing for meaningful comparisons between adolescent and adult athletes in both research and practical applications. Measurement invariance analysis provided compelling evidence that the Turkish version of the MFs functions equivalently across adolescent and adult athletes. MG-CFA confirmed support for configural, metric, and scalar invariance, with minimal deterioration in fit indices across nested models (ΔCFI ≤ 0.01; ΔRMSEA ≤ 0.01; ΔSRMR ≤ 0.01). Notably, the Comparative Fit Index (CFI) remained stable at 0.88 across the configural and scalar models, while the Tucker–Lewis Index (TLI) showed a slight improvement from 0.86 to 0.88. The RMSEA decreased from 0.08 to 0.07, and the SRMR remained constant at 0.06. Furthermore, strict invariance—by constraining residual variances—was achieved with an acceptable fit (CFI = 0.88, RMSEA = 0.07), and the structural model constraining factor variances also demonstrated comparable fit indices. These findings indicate that the scale’s factor structure, item loadings, intercepts, residual variances, and latent factor variances are equivalent between age groups, suggesting that variations in observed MFs scores reflect authentic differences in mental fatigue rather than measurement bias related to age.

### 4.1. Practical Applications

Turkish MFs may serve as a practical monitoring tool for sports scientists and coaches to identify mental fatigue in athletes and inform decisions regarding training load management, recovery strategies, and competition readiness. Situating these results within the sports psychology literature does not imply that MFs replace established monitoring tools. Rather, Turkish MFs may serve as a complementary instrument alongside state-based mental fatigue ratings, cognitive load measures, and workload indices, offering a comprehensive assessment of mental fatigue in athletes during training and competition periods. Beyond psychometric properties, these results have significant practical implications in sports settings. Turkish MFs provide coaches and support staff with a valid tool to monitor athletes’ subjective mental fatigue levels, which is crucial for optimizing training loads and preventing non-functional overreaching. Specifically, this scale can be integrated into daily monitoring routines alongside physiological markers (e.g., RPE and heart rate) to identify athletes who may be experiencing cognitive or sensory symptoms of fatigue that are not captured by traditional physical metrics. Early detection of high mental fatigue scores (>10.5) allows for timely adjustments in training intensity or implementation of recovery strategies.

### 4.2. Strength and Limitations

The strengths of this study include a large heterogeneous athletic sample examining both adolescent and adult athletes and a comprehensive psychometric evaluation of the factor structure, consistency, stability, and measurement invariance across age groups. These features enhance the generalizability of the findings to athletic populations. However, it has some important limitations. First, convergent, discriminant, and criterion validity were not examined because of the lack of external measures of mental fatigue, cognitive load, or performance outcomes. Thus, the findings represent factorial validity and reliability, rather than comprehensive construct validity. Second, measurement invariance was supported across adolescent and adult athletes but not across gender. Future studies should examine scale equivalence across genders and developmental stages. Third, the sample excluded clinical populations for whom the scale was developed. The applicability of Turkish MFs in clinical settings remains untested. Finally, self-reported data may have introduced response bias. Future research should combine self-reports with cognitive tasks, physiological markers, and mental fatigue performance indicators.

## 5. Conclusions

The present study provides evidence supporting the factorial structure and reliability of the Turkish version of the MFs in adolescent and adult athletes. Psychometric findings showed satisfactory internal consistency, temporal stability, and measurement invariance across age groups in this study. However, further evidence is needed before strong claims regarding construct validity can be made in this regard. Future research should examine convergent, discriminant, and criterion validity using external measures of mental fatigue and performance in a larger sample. Despite these limitations, the Turkish MFs appears promising for assessing mental fatigue in athletic populations, warranting continued validation.

## Figures and Tables

**Table 1:** Descriptive Statistics of the study participants (N = 491).

Variable	Mean	Std. Deviation	Skewness	Kurtosis	Minimum	Maximum
Age (year)	18.88	5.50	1.1	1.95	10	52
Height (cm)	171.35	10.42	−0.47	0.37	128	195
Weight (kg)	62.98	11.96	0.19	0.87	25	115
BMI (kg·min^−2^)	21.94	2.66	0.79	2.56	13.9	35.49
Athletic Experience (year)	7.05	4.27	0.81	0.66	0	23
MF_1	1.15	0.83	0.64	−0.05	0	3
MF_2	0.73	0.75	0.97	0.39	0	3
MF_3	0.70	0.75	0.99	0.44	0	3
MF_4	0.81	0.87	1.02	0.28	0	3
MF_5	0.88	0.78	0.99	0.96	0	3
MF_6	0.60	0.77	1.28	0.94	0	3
MF_7	0.47	0.59	1.45	2.45	0	3
MF_8	0.95	0.91	0.99	0.10	0	3
MF_9	0.78	0.89	1.25	0.85	0	3
MF_10	1.15	0.86	0.55	−0.31	0	3
MF_11	0.36	0.68	2.20	4.63	0	3
MF_12	0.73	0.77	1.11	1	0	3
MF_13	0.72	0.79	1.07	0.58	0	3
MF_14	0.54	0.82	1.68	2.02	0	3
MF_15	0.58	0.62	0.55	−0.6	0	2

MF = Mental fatigue; BMI = body mass index.

**Table 2:** Unidimensional Reliability (N = 491).

Estimate	McDonald’s ω	Cronbach’s α
Point estimate	0.88	0.88
95% CI lower bound	0.87	0.87
95% CI upper bound	0.90	0.90
MF_1	0.87	0.87
MF_2	0.87	0.87
MF_3	0.87	0.87
MF_4	0.88	0.87
MF_5	0.87	0.87
MF_6	0.88	0.87
MF_7	0.88	0.88
MF_8	0.88	0.87
MF_9	0.88	0.87
MF_10	0.88	0.87
MF_11	0.88	0.88
MF_12	0.88	0.87
MF_13	0.88	0.88
MF_14	0.88	0.88
MF_15	0.88	0.87

MF: Mental fatigue.

**Table 3:** Confirmatory Factor Analysis (CFA) of the Turkish MFs (N = 491).

Fit indices	Value
Comparative Fit Index (CFI)	0.89
Tucker–Lewis Index (TLI)	0.87
Bentler-Bonett Non-normed Fit Index (NNFI)	0.87
Bentler-Bonett Normed Fit Index (NFI)	0.85
Parsimony Normed Fit Index (PNFI)	0.73
Bollen’s Relative Fit Index (RFI)	0.83
Bollen’s Incremental Fit Index (IFI)	0.89
Relative Noncentrality Index (RNI)	0.89
Log-likelihood	−7501.04
Number of free parameters	45
Akaike (AIC)	15,092.08
Bayesian (BIC)	15,280.92
Sample-size adjusted Bayesian (SSABIC)	15,138.09
Root mean square error of approximation (RMSEA)	0.08
RMSEA 90% CI lower bound	0.07
RMSEA 90% CI upper bound	0.09
RMSEA *p*-value	1.52 × 10^−7^
Standardized root mean square residual (SRMR)	0.05
Hoelter’s critical N (α = 0.05)	159.22
Hoelter’s critical N (α = 0.01)	174.56
Goodness of fit index (GFI)	0.94
McDonald fit index (MFI)	0.77
Expected cross validation index (ECVI)	0.90

**Table 4:** Parameter estimates of the Turkish MFs (N = 491).

Item	Loading	Std. Loading	Std. Error	z-Value	*p*	95% Confidence Interval (LL)	95% Confidence Interval (UL)
MF_1	0.55	0.67	0.03	15.94	<0.001	0.48	0.62
MF_2	0.49	0.66	0.03	15.85	<0.001	0.43	0.56
MF_3	0.48	0.64	0.03	15.26	<0.001	0.42	0.54
MF_4	0.52	0.60	0.04	13.96	<0.001	0.45	0.59
MF_5	0.53	0.69	0.03	16.53	<0.001	0.47	0.60
MF_6	0.45	0.59	0.03	13.65	<0.001	0.39	0.52
MF_7	0.33	0.56	0.03	12.86	<0.001	0.28	0.38
MF_8	0.54	0.59	0.04	13.76	<0.001	0.47	0.62
MF_9	0.51	0.57	0.04	13.16	<0.001	0.43	0.58
MF_10	0.52	0.61	0.04	14.08	<0.001	0.45	0.59
MF_11	0.33	0.49	0.03	10.95	<0.001	0.27	0.39
MF_12	0.44	0.57	0.03	13.1	<0.001	0.37	0.50
MF_13	0.33	0.42	0.04	9.32	<0.001	0.26	0.40
MF_14	0.35	0.43	0.04	9.38	<0.001	0.28	0.42
MF_15	0.37	0.61	0.03	14.17	<0.001	0.32	0.43

MF: Mental fatigue. Std. Loading: Standardized loading.

## Data Availability

The original data presented in this study are openly available in Zenodo at https://doi.org/10.5281/zenodo.17761951.

## Abbreviations

The following abbreviations are used in this manuscript:

MF	Mental Fatigue
MFs	Mental Fatigue Scale
fNIRS	Functional Near-infrared Spectroscopy
EEG	Electroencephalogram
BRUMS	Brunel Mood Scale
MG-CFA	Multi-group confirmatory factor analysis
VAS	Visual Analogue Scale
ICC	Intra-class Correlation Coefficient
CFA	Confirmatory Factor Analysis
SEM	Structural Equation Modelling
TLI	Tucker–Lewis index
SRMR	Standardized Root Mean Square Residual
NNFI	Bentler-Bonett Non-normed Fit Index
NFI	Bentler-Bonett Normed Fit Index
CI	Confidence Interval
F-ISA	Fatigue Instantaneous Self-Assessment
